# DeepMap+: Recognizing High-Level Indoor Semantics Using Virtual Features and Samples Based on a Multi-Length Window Framework

**DOI:** 10.3390/s17061214

**Published:** 2017-05-26

**Authors:** Wei Zhang, Siwang Zhou

**Affiliations:** College of Computer Science and Electronic Engineering, Hunan University, Changsha 410000, China; zweihnu@hnu.edu.cn

**Keywords:** indoor semantic inference, activity recognition, multi-length windows, virtual samples, virtual features, deep learning

## Abstract

Existing indoor semantic recognition schemes are mostly capable of discovering patterns through smartphone sensing, but it is hard to recognize rich enough high-level indoor semantics for map enhancement. In this work we present DeepMap+, an automatical inference system for recognizing high-level indoor semantics using complex human activities with wrist-worn sensing. DeepMap+ is the first deep computation system using deep learning (DL) based on a multi-length window framework to enrich the data source. Furthermore, we propose novel methods of increasing virtual features and virtual samples for DeepMap+ to better discover hidden patterns of complex hand gestures. We have performed 23 high-level indoor semantics (including public facilities and functional zones) and collected wrist-worn data at a Wal-Mart supermarket. The experimental results show that our proposed methods can effectively improve the classification accuracy.

## 1. Introduction

As people spend the majority of their time in indoor environments [1], indoor environment inference plays an increasing significant role in pervasive and mobile computing. Indoor location-based services (LBSs) are developed to greatly impact on human life and enable various novel indoor applications, such as indoor positioning [2], vehicle navigation [3], user tracking [4] and so on [5]. For the majority of indoor LBSs, the most significant foundation is the indoor map [6,7,8,9,10]. A useful indoor map contains both spatial information (such as the structure and the size of indoor floor) as well as crucial landmark map semantics such as emergency exits, elevators, doors, washrooms, etc. Important indoor semantics can greatly enrich indoor maps and better guide persons to their destinations. In recent years, indoor semantic recognition has been received much attention from researchers. For example, Jigsaw [8] achieved the extraction of geometric features of individual landmarks from images. However, geometric features are the low-level semantics of the indoor environment, and a complete floor map needs to contain high-level semantic elements. Cheng [9] presented iMap, a high-level semantic inference system, which can automatically detect four different indoor semantics: stairs, elevators, elevators and doors. Nevertheless, it could not provide enough indoor semantics for map enhancement. It is very difficult to recognize abundant high-level semantics because the researchers can hardly discover most of their patterns with mobile device sensing. To overcome the problem, authors of [10] designed TransitLabel, which used passengers’ activities to infer indoor semantics in a digital map, so it can recognize 19 high-level transit station semantics. Although TransitLabel has already made a remarkable achievement in indoor semantic recognition, it is still imperfect and this paper has three overarching challenges as follows:

(1) Due to the large number of indoor semantics in some important indoor public areas such as supermarket and so on, there inevitably exist similar interaction activities between each user and different indoor facilities. It is hard to recognize the similar activities for inferring various indoor semantics. In particular, most of the interaction activities should have much more fine-grained and complex hand gestures in these indoor public areas than in activities of daily living (ADLs). For example, the hand gestures of taking a sandwich by using a bread tong are very similar to the movements of ladling out rice with a measuring cup. The above two activities can be utilized to infer the bread counter and the rice storage shelf, respectively. Therefore, it is necessary that the classifier system have an improved discriminating power for the fine hand movements.
(2) To improve the classification accuracy of high-level indoor semantics, an effective way is by increasing the number of sensors, collected samples or extracted features, but all of them would burden the power constrained by mobile device and impact user comfort.
(3) TransitLabel [10] enables automatic inference of high-level indoor semantics relying on a tree structure with some prior knowledge (such as vertical speed threshold and altitude threshold, etc.). The tree structure of TransitLabel decides that every inference of indoor semantics is dependent. However, as we know, indoor semantics are being updated and indoor mobile sensing (such as air pressure, audio and so on) is highly susceptible to wild fluctuations in accuracy when used in diverse indoor environments. In our opinion, the above prior knowledge is not absolutely reliable and the tree structure is not beneficial for the dynamic update of the inference system, so it is necessary to make the inference system more intelligent.

To overcome these challenges, we pay more attention to human gesture recognition since significant indoor facilities are closely relative to complex human gestures. Firstly, we attempt to bridge the gap between indoor semantic inference and wearable device sensing. The smart watch is chosen as data collection device, because wrist-worn sensing is extremely beneficial for capturing human activities. In addition, we design a high-level indoor semantic inference for recognizing more abundant high-level indoor semantics than in the literature [8,9]. It is used to infer indoor semantics from users’ complex activities and users’ location contexts instead of conventional location sensors such as GPS, Bluetooth beacons and so on. Furthermore, for fine activity recognition, we propose a novel multi-length window framework instead of the single-length window framework which is widely used in pattern recognition. Our novel idea is illustrated by experiment 1 described in Section 5.3. There are two important gains, summarized as follows: (1) The length of the sliding windows can greatly affect the complex activity recognition. We find that the single-length window framework is enough for simple activities but may not be sufficient for complex activities such as taking off jackets, putting on shoes and so on. That is because diverse patterns of various complex activities lie in different-length windows; and (2) We find out the existing characteristics and correlations between the different-length windows. By further analysis, we know that there exist different characteristics between the same features as well as cross correlations between the same classes from different-length windows. Both of them are beneficial to generate virtual features and samples which could strengthen the activity recognition ability and further improve the classification accuracy of indoor semantics. In particular, our proposed methods would not burden the mobile device and the generated virtual features and samples can effectively improve discriminative ability of our system for high-level indoor semantics.

The outcome of our investigation is DeepMap+—A data-driven system for automatically recognizing high-level indoor semantics with wrist-worn sensing. It only utilizes three sensors such as an accelerometer, a gyroscope and an air pressure sensor. DeepMap+ can automatically learn robust representations by DL algorithm from a new synthetic training dictionary containing virtual features and samples. These representations are obtained in a dense inter-connected network of units, and each unit has a relatively simple function parameterized by whole training data. In summary, this paper makes the following contributions:

(1) We present DeepMap+, which is the first deep computation model based on the multi-length window framework for recognizing high-level indoor semantics using complex human activities. Instead of the conventional single-length window framework, the multi-length window framework can greatly enrich our data storage. In addition, we design a high-level indoor semantic inference to infer users’ location contexts and high-level indoor semantics (consisting of public facilities and functional zones) at a Wal-Mart supermarket.
(2) We discover the characteristics and the correlations between the different-length windows and find out that their properties are beneficial to human activity classification. Based on this, for finer grained activity recognition we propose several methods of increasing virtual features and samples which are helpful to generate a valuable synthetic training dictionary. By integrating the deep learning (DL) technique, DeepMap+ can learn robust representations from the synthetic training dictionary.
(3) We implement an Android application for the mobile client and Python program that runs on the server side. The Android application is developed for wrist-worn sensing, and a deep neural network (DNN)-based classifier is trained and its parameters are tuned with supervised learning.
(4) We conduct performance validation with an exhaustive experimental study consisting of wrist-worn data collection of 23 high-level indoor semantics by two users at a Wal-Mart supermarket.

The rest of this paper is organized as follows. Section 3 presents the system overview of DeepMap+ and a high-level indoor semantic inference. We introduce the novel methods of increasing virtual features and samples in Section 4. Section 5 presents detailed experimental evaluation and validation of DeepMap+. Finally, Section 6 concludes this work with a discussion on future works.

## 2. The Related Work

In recent years, map semantic inference has received much attention from researchers. For example, Map++ [11] automatically identified different road semantics to enrich digital maps. However, this is an out-door map semantics-identified system. Jigsaw [8] was presented as a in-door floor plan reconstruction system through leveraging crowd-sensed data from mobile users. It achieved in extracting geometric features of individual landmarks from images. However, geometric features are the low-level semantic of the indoor environment, and a complete floor map needs to contain high-level semantic elements such as doors, washroom, escalator, stairs, etc. Then, Cheng [9] presented iMap, which was a high-level semantic inference system for automatically annotating the indoor maps. It detected four different indoor semantics: stairs, elevators, escalators and doors. Nevertheless, a useful indoor map needs rich enough high-level fine-grained semantics. Elhamshary et al. [10] designed TransitLabel, which used passengers’ activities to infer indoor semantics on a digital map, and it can recognize 19 high-level transit station semantics. TransitLabel has already made a remarkable achievement in indoor semantic recognition, but these authors [10] have not performed further research on human activities; it may have a negative influence on the recognition accuracy of indoor semantics.

Most of existing works on indoor activity recognition focus on in-home elderly healthcare problems [12,13] and physical activity monitoring problems [14,15]. They always perform the research of ADLs recognition, but the complexity of ADLs is seriously limited in the range of human daily life. Yan et al. [16] designed a 2-tier activity extraction framework to detect six activities at home and six activities at the office. Wang et al. [17] proposed CARM, which is a human activity recognition and monitoring system with a channel state information (CSI) signal. Its advantage was device-free for users, but it only recognized nine simple activities such as running, walking and sitting down, etc. Similarly, the studies [14,15,18] did not pay enough attention to this problem. As wearable device sensing is increasingly applicable for activity recognition [19], the problem is alleviated gradually. De et al. [12] utilized several wearable sensors on multiple body positions to recognize 19 fine-grained in-home activities. Now, the number of indoor activities for classification has increased greatly, but it cannot fundamentally improve with respect to the complexity of activities such as lying, sitting, walking and so on.

Recently, as a new direction developed rapidly, the deep neural network (DNN) has promoted a speedy advance in many fields such as speech recognition [20], visual object recognition [21], object detection [22] and so on [23,24]. Its main characteristic is deep structured learning through leveraging large-scale datasets. As we know, A-Wristocracy [13] is the first deep learning neural network-based activity classifier, and it is able to recognize fine-grained 22 daily activities with high average test accuracy. Unfortunately, the complexity of above daily activities is also limited by human basic daily actions.

Inevitably, there always exist complex interaction activities between users and significant indoor infrastructures in real life. In our opinion, we should strengthen human gesture recognition because the complex interaction activities should have rich hand movements. In this paper, we try to bridge the gap between indoor semantic inference and the wrist-worn sensing, and we hold that the self-learning ability of DL can motivate the field of indoor semantic recognition. In addition, we note that recent studies [9,10,12] have utilized more and more environmental sensors like temperature sensors, humidity sensors, magnitude sensors and other location sensors. Undoubtedly, more sensors can efficiently improve the classification accuracy of indoor semantics, but it is easy to put additional burdens on the measurement device. Unlike earlier works, this paper attempts to recognize high-level indoor semantics and simultaneously tries to enhance hand gesture recognition for avoiding the utilization of unnecessary sensors.

## 3. The DeepMap+ System

In this section we firstly introduce the architecture of DeepMap+, and then present the feature extraction and the deep learning model of DeepMap+. In the end of the section, we further illustrate our designed high-level indoor semantic inference for DeepMap+ at a Wal-Mart supermarket.

### 3.1. Overview

**Figure 1:**
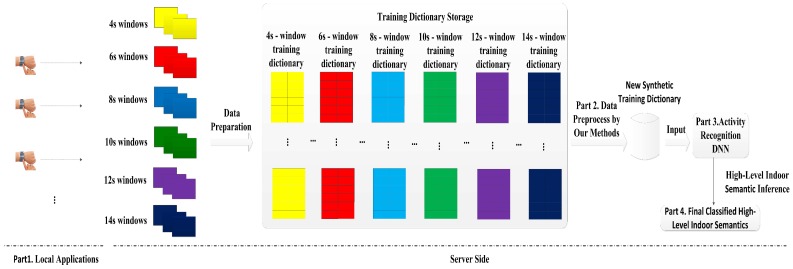
The DeepMap+ system architecture. DNN: Deep neural network.

DeepMap+ is designed for automatically recognizing high-level indoor semantics with users’ wrist-worn sensing at a Wal-Mart supermarket. Figure 1 presents the DeepMap+ architecture which consists of four parts. In the first part, we develop an Android application for: (1) stopping and starting the wrist-worn data collection; (2) manually inputting the ground truth information with proper timestamp; and (3) uploading the collected data and labels. In the second part, multi-length sliding windows of 4, 6, 8, 10, 12 and 14 s are generated using raw data. Meanwhile, their corresponding training dictionaries are obtained and stored in the data storage. Our proposed methods preprocess above training dictionaries of multi-length windows and transform them into virtual features and virtual samples, which are stored for generating virtual feature matrix and virtual sample matrix. The third part of DeepMap+ is DL [23] with a new synthetic training dictionary for complex activity classification. The new synthetic training dictionary is utilized by DNN for automatically learning more robust representations compare to the original training dictionary, because it contains not only the original training dictionary but also the generated virtual feature matrix and virtual sample matrix. Our proposed methods for generating the virtual feature matrix, the virtual sample matrix and the synthetic training dictionary are discussed in details in the next section. The last part is a high-level indoor semantic inference with complex human activities for the Wal-Mart supermarket.

### 3.2. Feature Extraction

DeepMap+ attempts to not damage the user-friendliness of the measurement device, so we drop the advanced multi-modal sensing [10,12,25] to free the mobile device from overloaded sensors although multi-sensors can bring us useful environment contexts. For easing the burden of mobile device, we just utilize inertial sensors such as an accelerometer, a gyroscope and an air pressure sensor to recognize complex activities. The accelerometer and gyroscope features are designed as in [13] and sampled at 100 Hz, including six accelerometer features and six gyroscope features. These accelerometer and gyroscope features are designed as follows: mean and variance of resultant acceleration ($\sqrt{a_{x}^{2}+a_{y}^{2}+a_{z}^{2}}$ where $a_{x},a_{y}$ and $a_{z}$ are the three axis of acceleration), mean and variance of resultant angular speed ($\sqrt{b_{x}^{2}+b_{y}^{2}+b_{z}^{2}}$ where $b_{x},b_{y}$ and $b_{z}$ are the three axis of angular speed), mean and variance of first derivative of resultant acceleration, mean and variance of first derivative of resultant angular speed, mean and variance of second derivative of resultant acceleration, and mean and variance of second derivative of resultant angular speed. In addition, the air pressure sensor is applied to sample the atmospheric pressure at 5 Hz for recognizing the escalators and the stairs. The atmospheric pressure feature is the variance of per second data for air pressure sensor.

For DeepMap+, the air pressure sensor is enabled which is for two reasons: (1) the escalators and the stairs are significant indoor semantics; and (2) it fails to recognize them only with human activity features because the activities (walking and standing) are too common in the escalators and the stairs so that the atmospheric pressure feature should be sampled. Therefore, the air pressure sensor is indispensable as are the accelerometer sensor and the gyroscope sensor. We insist that DeepMap+ only enables the sensors which are indispensable for the recognized targets. For example, if we utilize a temperature feature sampled by the temperature sensor, it is easy to distinguish indoor semantics like a one-door soda fountain, a two-door upright freezer or a horizontal freezer from other indoor semantics in normal temperature, but we can also recognize above complex human activities without temperature sampling and finally infer the above three indoor semantics. From this point of view, we have made an attempt to make other environmental sensors like the temperature sensor, humidity sensor, and magnitude sensor unnecessary and disable them to save more time and resources, but a fewer number of sensors proposes a higher requirement for finely recognizing users’ hand gestures. Similarly to increasing sensors, increasing features should also add more burdens to low-power wearable devices [13]. DeepMap+ is also designed to not increase the number of samples and features collected from accelerometer and gyroscope sensors on the measurement device. However, we still need more information about the users’ hand gestures. We found that the multi-length window framework can retain much more important information compared to the conventional single-length window framework. Therefore, we built a data storage of multi-length windows for our system as illustrated in Figure 1. For better utilization of the data source of multi-length windows, we design several methods to obtain virtual samples and features for DeepMap+ to finely distinguish the hand gestures. Our designed methods are described in the next section.

### 3.3. Deep Learning-Based Activity Recognition

For classification-oriented problems, such as the complex activity recognition, the appropriate features are very significant for recognition accuracy [26]. Designing acceptable hand-crafted features requires expert knowledge and extensive experiments. The designed accelerometer features and gyroscope features by [13] are low-level features, and it is hard to discriminate human complex activities with low-level features. Therefore, high-level feature extraction is indispensable for DeepMap+. As an advanced feature extraction algorithm, deep learning has been applied in many fields of pattern recognition. As shown in Figure 1, DeepMap+ is the first deep computation model based on multi-length windows. In DeepMap+, a stacked autoencoder [27,28] is selected as our DL algorithm for complex human activity recognition. The stacked autoencoder is a neural network consisting of multiple layers of autoencoder (AE) [29].

**Figure 2:**
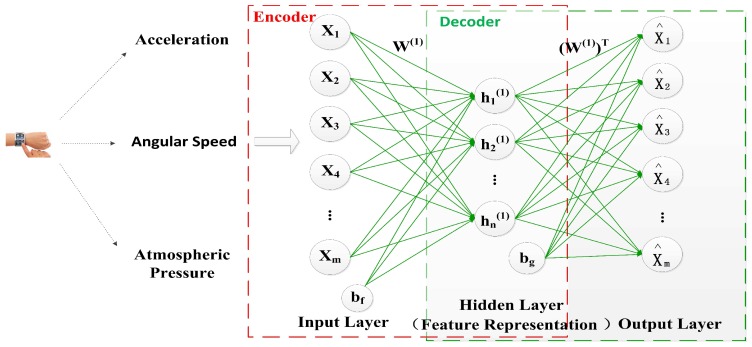
The architecture of the basic autoencoder.

The architecture of the basic AE is illustrated in Figure 2. The input data of the AE is the training dictionary $D={\left[x\left(1\right),x\left(2\right),\ldots ,x\left(N\right)\right]}^{T}$ which consists of *N* training samples, and each training sample is represented as an m-dimensional feature vector *x*. It is above low-level features about acceleration, angular speed and atmospheric pressure of user’s wrist. Therefore, we can get the encoder $h^{\left(1\right)}=f\left(x\right)=s_{f}\left(W^{\left(1\right)}x+b_{f}\right)$, where the parameters $W^{\left(1\right)}\in R^{n\times m}$ and $b_{f}\in R^{n}$. *m* is the dimensionality of the input *x* and *n* is the number of the units of the hidden layer. Then, the output of the encoder $h^{\left(1\right)}$ is used as the input of the decoder $\hat{x}=g\left(h^{\left(1\right)}\right)=s_{g}\left(W^{{\left(1\right)}^{T}}h^{\left(1\right)}+b_{g}\right)$, where the parameters $W^{{\left(1\right)}^{T}}\in R^{m\times n}$ and $b_{g}\in R^{m}$. Here $s_{f}$ and $s_{g}$ are ReLU activation functions of the encoder and the decoder respectively. For retaining the most information about input data, the AE aims to minimize the reconstruction error through finding the optimal $W^{\left(1\right)},W^{{\left(1\right)}^{T}},b_{f},b_{g}$ and it is given as follows:

(1) $$\min_{W^{\left(1\right)},W^{{\left(1\right)}^{T}},b_{f},b_{g}}\;\sum_{x\in D}L\left(x,\hat{x}\right)$$

Here the reconstruction error is the squared error $L\left(x,\hat{x}\right)=∥x-\hat{x}{∥}^{2}$.

**Figure 3:**
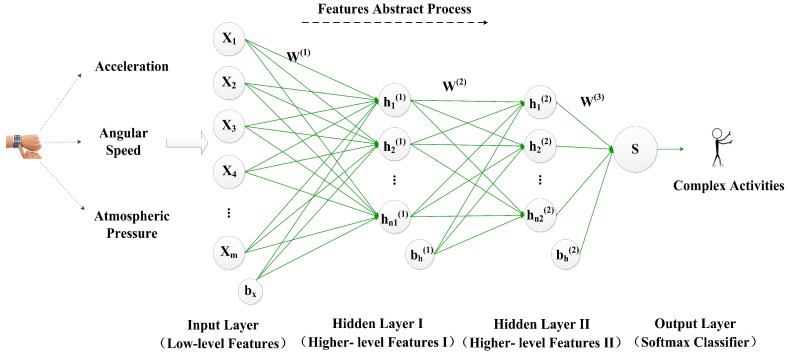
The architecture of the stacked autoencoder used in DeepMap+.

Figure 3 shows the architecture of the stacked autoencoder (SAE) in DeepMap+. This SAE consists of two hidden layers and represents the two-layer AE. For simplicity, we have not shown the decoder parts of each AE in Figure 3. In a manner similar to AE, after training the parameters of the first hidden layer, the output $h^{\left(1\right)}$ of the first hidden layer is the input of second hidden layer. The deeper layers progressively represent the inputs in a more abstract way, so more higher-level features can be extracted than the input data. In the output layer, the softmax classifier (SMC) [30] is utilized so that the DNN-based complex activity classifier is constructed. DeepMap+ uses the greedy layer-wise training [31] to obtain good parameters of each unit of the multi-layers; the training procedure includes the three steps as follows:

First, the SAE is applied to learn primary representation(high-level features I) $h^{\left(1\right)}\left(x\right)$ from the low-level features *x* by adjusting the weight $W^{\left(1\right)}$ and the bias $b_{x}$;

Second, above primary representation $h^{\left(1\right)}\left(x\right)$ is used as the input to the other autoencoder to learn the secondary representation (high-level features II) $h^{\left(2\right)}\left(x\right)$ on the primary representation by adjusting the weight $W^{\left(2\right)}$ and the bias $b_{h^{\left(1\right)}}$;

At last, the secondary representation $h^{\left(2\right)}\left(x\right)$ is treated as the input to the SMC, and it is trained to map $h^{\left(2\right)}\left(x\right)$ to digitally label *y* by adjusting the weight $W^{\left(3\right)}$ which is the parameter of the SMC model.

To get better parameters, DeepMap+ utilizes fine-tuning [24] which is based on the backpropagation algorithm during model training. Fine-tuning can improve the results by tuning the parameters of above all layers which are changed at the same time. Therefore, DeepMap+ can recognize complex human activities using higher-level features $h^{\left(2\right)}$ which are learned by the stacked autoencoder. From above deep learning process, we can find that the parameters of above all layers may be more appropriate, benefiting from the increased samples in the training process. Furthermore, we add more useful information to raw input data such as increasing the dimensionality (feature number) of the input data *x*, which is also of benefit to obtain a robust representation of the wrist-worn data for complex activity recognition in DeepMap+. Therefore, the multi-length window framework is applied to an extent in our dataset and we propose lots of methods for increasing some virtual samples and features based on this framework in Section 4.

### 3.4. High-Level Indoor Semantic Inference

**Figure 4:**
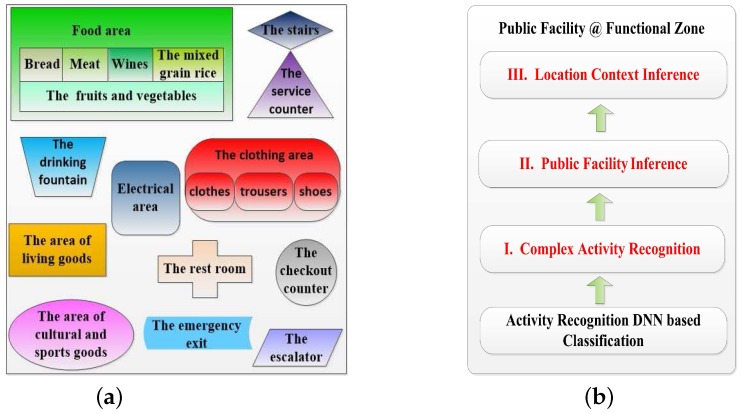
(**a**) The functional zones and separate facilities of the Wal-Mart supermarket; (**b**) high-level indoor semantic inference.

In this subsection, we design a high-level indoor semantic inference which makes sure that DeepMap+ can recognize abundant high-level indoor semantics at the Wal-Mart supermarket. As the indoor semantics are inferred by human complex activities in the fourth part of DeepMap+ as shown in Figure 1, DeepMap+ greatly extends the scope of indoor semantic recognition compared to [8,9]. Furthermore, the high-level indoor semantics for DeepMap+ include not only the public facilities but also the location contexts such as functional zones. We find out the public facilities of Wal-Mart are closely related to users’ activities and functional zones. At first, we exact significant functional zones and separate facilities of Wal-Mart illustrated in Figure 4a. They are important location contexts of the recognized targets for the users, and they themselves also are crucial indoor semantics of Wal-Mart. Then, we select several typical facilities as our recognized targets which are important indoor semantics. In Figure 4a, every functional zone has at least a significant facility so that each functional zones can be inferred by at least one recognized target. In addition, we make a correlation rule which can ensure the one to one mapping relationship between the facilities and the activities, as shown in Table 1. Table 1 also introduces the detailed description of each of the complex activities, respectively.

As shown in Figure 4b, DeepMap+ infers the final high-level indoor semantics of Wal-Mart with the high-level indoor semantic inference. The first layer is the complex activity recognition with supervised DL. The second layer is the public facility inference based on complex activities and the third is the location context inference based on typical facilities in accordance with the correlation rule in Table 1. To some extent, the third layer infers users’ location contexts instead of location sensors such as GPS, Bluetooth beacons and so on. Therefore, the final high-level indoor semantic is equal to a public facility at the functional zone. For example, DeepMap+ recognizes a customer’s activity such as trying on the shoes, meanwhile it can infer a shoe cabinet in the shoes section of the clothing area in Wal-Mart.

It should be noted that the inference of each high-level indoor semantics is independent without prior knowledge due to the DL process, and we only need to update Table 1 if any indoor semantics are changed at Wal-Mart. Furthermore, our biggest challenge is shifted from high-level indoor semantic inference to complex activity recognition, and most of the complex activities have several fine-grained hand movements as shown in Table 1. Especially, some hand gestures of them are very similar, such as filling cereal food into a storage bag and bagging bulk food in the food area, etc. To solve above problems, the number of features and samples is particularly significant when the number of sensors is not changed.

## 4. The Description of Our Proposed Methods

To strengthen the fine-grained recognition of complex activities, this section describes how we generate virtual features and virtual samples based on the training dictionaries of multi-length windows from the data storage of DeepMap+. Let *k* denote the number of activity classes (*k* is equal to 23 in this paper) and $n_{i}$ denote the number of training samples from *i*-th class, i∈[1,2,…,k]. Each training sample is represented as an *m*-dimensional feature vector (*m* is equal to 13 in this paper). We arrange the $n_{i}$ training samples from class *i* as rows of a data matrix $D_{i}={\left[x_{i,1},x_{i,2},\ldots ,x_{i,n_{i}}\right]}^{T}\in \;R^{n_{i}\times m}$. In this paper, $D^{4s}$, $D^{6s}$, $D^{8s}$ and $D^{10s}$ represent 4-, 6-, 8-, and 10-s window training dictionaries, respectively. In the conventional single-length window framework, $D^{4s}$ is the original training dictionary if it adopts the sliding windows with a length of 4 s, $D^{6s}$ is the original training dictionary when adopting the sliding windows with a length of 6 s, and so on. In our designed multi-length window framework, the above training dictionaries are generated with 50% overlapped sliding windows which are derived from the same data source sampled by users per second as described in Section 3.2. Each of the training dictionaries have the same dimensionality *m*, and the features of them are arranged in the same order. However, the training dictionaries have different numbers of rows because the different lengths of the sliding windows result in the different number of total samples. For enhancing the original training dictionary, we design lots of methods to add virtual features or virtual samples with a supplemental dictionary from the data source of multi-length windows. For better description of our proposed methods, we firstly illustrate the process of data preparation in the data storage of DeepMap+.

### 4.1. The Preprocess of the Supplemental Dictionary

**Figure 5:**
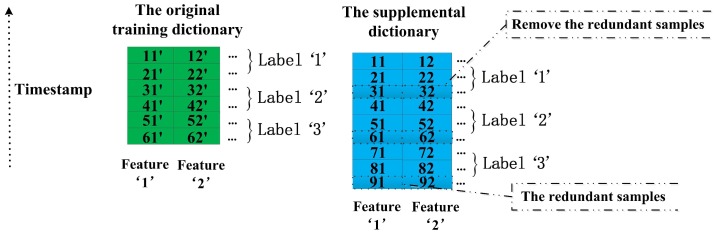
The preprocess of the supplemental dictionary.

This subsection introduces the important details about how to preprocess the supplementary dictionary in the process of data preparation. As we described in the beginning of this section, the training dictionaries $D^{4s}$, $D^{6s}$, $D^{8s}$ and $D^{10s}$ have the same number of columns but a different number of rows. Obviously, we can see $C_{D^{4s}}>C_{D^{6s}}>C_{D^{8s}}>C_{D^{10s}}$, and $C_{D^{4s}}$ denotes the sample number of $D^{4s}$. This is one of significant reasons why we choose the training dictionary of shorter-length windows as the supplementary dictionary for the original training dictionary and there is no need to worry about the lack of the supplementary data. The other advantages of this method are illustrated in the following Section 4.5. As shown in the Figure 5, we list a simple example for the preprocess of the supplemental dictionary. In the preprocess, we transform the supplemental dictionary to achieve two targets: (1) the structure of the supplemental dictionary is as the same as the original training dictionary; and (2) the property of the supplemental dictionary is close to the original training dictionary. At first, all entries of each classes of the original training dictionary and the supplemental dictionary are sorted by their timestamp in descending order. For the original training dictionary in the Figure 5, the entries 11${}^{\prime }$, 12${}^{\prime }$ denote the first samples of the first feature and the second feature respectively, and they are labeled as class ‘1’. We can see that the entries 11, 12 of the supplemental dictionary should be related with the entries 11${}^{\prime }$, 12${}^{\prime }$ of the original training dictionary in the time domain because they are labeled as the same class and derived from the same data source in the similar period. Similarly, the other entries of the original training dictionary also have the related entries in the supplemental dictionary. In Figure 5, the entries 21’, 22’, 31’, 32’, 41’, 42’, 51’, 52’, 61’, 62’ of the original training dictionary correspond to the entries 21, 22, 41, 42, 51, 52, 71, 72, 81, 82 of the supplemental dictionary, respectively. In addition, we need to remove the redundant samples of each class of the supplemental dictionary. It is easy to perform because we just need to remove the rows of the redundant samples. Respectively, the third, sixth and ninth rows of the supplemental dictionary are redundant for class ‘1’, class ‘2’ and class ‘3’ corresponding to the original training dictionary. Therefore, the third, sixth and ninth rows of the supplemental dictionary are deleted. Finally, the structure of the original training dictionary and the structure of the supplemental dictionary are the same totally due to their same dimensionality *m* and their same order of features arrangement. In all examples of this section, the sample number of the original training dictionary and its supplemental dictionary is six and the number of classes is three for convenience. Actually, the number of real samples is much higher than six and the number of classes is 23 in this paper. Now, we continue to introduce each of our proposed methods in the following sections.

### 4.2. The Methods of Increasing Features

The methods of increasing features are used to add diversity of discriminative features which can better characterize the hidden patterns of hand gesture. For example, a 4-s window feature and a 6-s window feature (such as mean of resultant acceleration $\sqrt{a_{x}^{2}+a_{y}^{2}+a_{z}^{2}}$) could be regarded as two different features that seize the various characters in their own windows. Although the training dictionary $D^{6s}$ and its supplemental dictionary $D^{4s}$ are correlated in time-domain, there still exist some different characteristics between the 4-s window features and 6-s window features. The different characteristics are derived from the different lengths of windows and confirmed in our experiment 1 described in Section 5.3, and we use the characteristics to generate the virtual features.

#### 4.2.1. The Method of Double-Length Window Features

**Figure 6:**
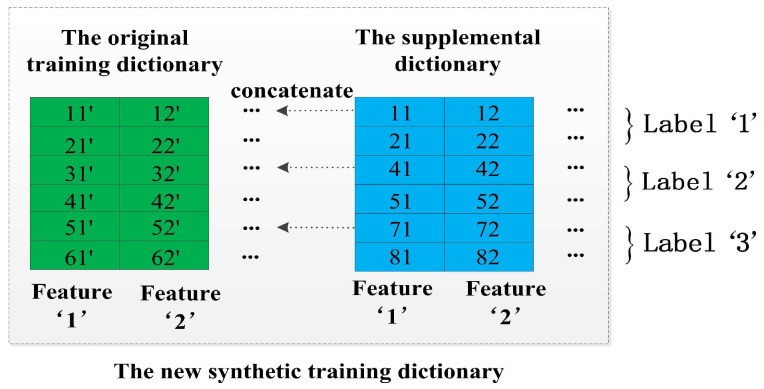
The method of double-length window features.

The core idea of this method is utilizing an original training dictionary and an additional dictionary based on shorter length windows to double the number of features for better capturing the essence of activities. For example, we can view an 8-s window training dictionary $D^{8s}$ as a supplementary of the original training dictionary $D^{10s}$ and concatenate them horizontally. Figure 6 shows a simple example of this method: the feature ’1’ of the original training dictionary $D^{origin}$ and the feature ’1’ of its supplemental dictionary $D^{supply}$ are viewed as two different features, and we finally obtain a new synthetic training dictionary $D^{synthetic}$ which has a double dimensionality than the original training dictionary $D^{origin}$.

Similarly, the methods of increasing virtual features are designed to double the dimensionality of the training dictionary as the method of double-length window features, but they generate a virtual feature matrix instead of the supplemental dictionary. The advantage is that the virtual feature matrix inherits various properties of multi-length windows, and we try to utilize the above properties for boosting the classification ability of DeepMap+. Now, we continue to introduce the methods of increasing virtual features.

#### 4.2.2. Increasing Virtual Features Based on Double-Length Windows

**Figure 7:**
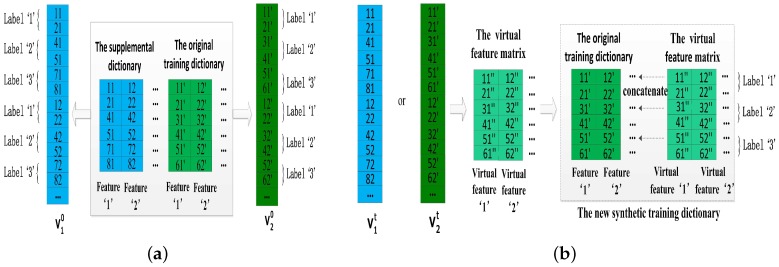
The method of increasing virtual features based on double-length windows. (**a**) Step 1: converting the original training dictionary and its supplemental dictionary to the vectors; (**b**) Step 2: obtaining virtual feature matrix *F* by gradient descent and horizontally concatenating it with the original training dictionary.

This method is also based on an original training dictionary and its supplemental dictionary from double-length windows (e.g., 10-s windows and 8-s windows). A simple example of the method is illustrated in Figure 7a,b, the main steps could be summarized as follows.

Step 1: Converting the original training dictionary $D^{origin}$ and its supplemental dictionary $D^{supply}$ to the vectors $v_{1}$ and $v_{2}$, respectively. Due to the same structure of $D^{origin}$ and $D^{supply}$, the lengths of $v_{1}$ and $v_{2}$ should be same.

Step 2: Using the gradient descent algorithm to obtain a virtual feature matrix *F* and horizontally concatenate it with $D^{origin}$ to generate a new synthetic training dictionary $D^{synthetic}$.

As shown in Figure 7a, let $v_{1}$ and $v_{2}$ represent the vector of $D^{supply}$ and $D^{origin}$, respectively. The goal of our method is to obtain a virtual feature matrix *F* instead of the supplemental dictionary $D^{supply}$ and *F* can inherit various properties of $D^{origin}$ and $D^{supply}$. Obviously, the vector form of *F* is approximately equal to $v_{1}$ and $v_{2}$. Therefore, we choose the gradient descent algorithm to update above $v_{1}$ and $v_{2}$, and $v_{1}^{0}$ and $v_{2}^{0}$ denote their initial values. The gradient descent function f(x) is:

(2) $$x^{t+1}=x^{t}-\alpha ▽f\left(x^{t}\right)$$

where α denotes the learning rate, and $x^{t}$ represents the value of *x* at time *t*. The minimum value of f(x) and the corresponding optimal value of *x* can be efficiently solved by the gradient descent algorithm. Then, our problem could be formulated as a simple function $L\left(v_{1},v_{2}\right)=∥v_{1}-v_{2}{∥}_{2}^{2}$, and $L(v_{1},v_{2})$ reaches its minimum value which means that we get approximately equal $v_{1}$ and $v_{2}$. It is easy to get ${▽}_{v_{1}}L\left(v_{1},v_{2}\right)=2\left(v_{1}-v_{2}\right)$ and ${▽}_{v_{2}}L\left(v_{1},v_{2}\right)=2\left(v_{2}-v_{1}\right)$. To iteratively update $v_{1}$ and $v_{2}$, the functions are:

(3) $$v_{1}^{t+1}=v_{1}^{t}-\alpha \left(v_{1}^{t}-v_{2}^{t}\right)$$

(4) $$v_{2}^{t+1}=v_{2}^{t}-\alpha \left(v_{2}^{t}-v_{1}^{t}\right)$$

where t is the number of iterations, $\alpha =\frac{\lambda }{t}$ and λ is a positive constant. As we know, the solution would achieve convergence as α decreases with the increase of *t*. Therefore, the optimal vector $v_{1}^{t}$ or $v_{2}^{t}$ is converted to the virtual feature matrix *F* which has the same structure as the original training dictionary. Finally, we could concatenate the original training dictionary $D^{origin}$ with virtual feature matrix *F* to obtain the final synthetic training dictionary $D^{synthetic}$.

#### 4.2.3. Increasing Virtual Features Based on Multi-Length Windows

**Figure 8:**
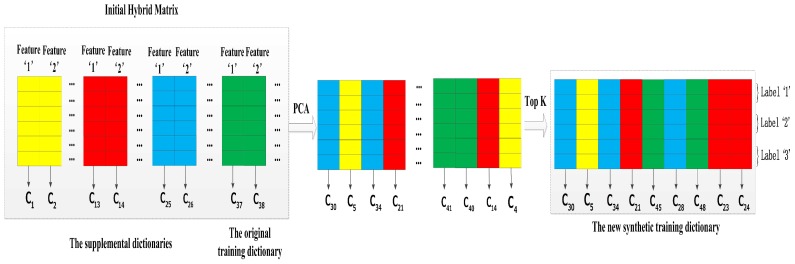
The method of increasing virtual features based on multi-length windows.

In an attempt to catch more useful information from multi-length windows, we regard each features of all training dictionaries as independent individuals. From this opinion, we extend the utilized range of supplemental dictionaries and propose a method of increasing virtual features based on multi-length windows. Therefore, its advantage is that it has several supplemental dictionaries and the methods based on double-length windows only have one supplemental dictionary. For example, we select the 10-s window training dictionary as the original training dictionary, and all of the 4-, 6-, and 8-s window training dictionaries become its supplemental dictionaries in this method. Figure 8 presents this example of the method, and we summarize the main steps as follows:

Step 1: Concatenating $D^{origin}$ with its supplemental dictionaries which based on shorter length windows to obtain a initial hybrid matrix.

Step 2: Recombining the hybrid matrix by PCA that could automatically discover the cross correlations which characterize the activity difference.

Step 3: Extracting the first *K* PCA components and discarding the rest to remove the poor quality features, and the first *K* PCA components constitute our synthetic training dictionary $D^{synthetic}$.

Before performing Step 1, the column of atmospheric pressure feature is removed in all dictionaries at first. After performing the Step 3, we add the column of atmospheric pressure feature of $D^{origin}$ to $D^{synthetic}$ so that it ensures the synthetic training dictionary $D^{synthetic}$ containing an atmospheric pressure feature. At last, the final dimensionality of the synthetic training dictionary $D^{synthetic}$ is equal to K+1.

### 4.3. The Method of Increasing Virtual Samples

**Figure 9:**
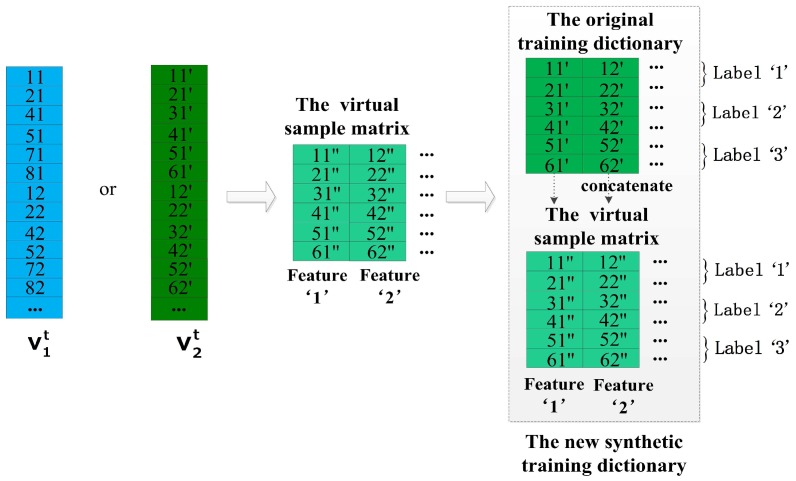
The method of increasing virtual samples: Step 2.

The method of increasing virtual samples confirms that cross correlation exists between the same classes from double-length windows (e.g., 10-s windows and 8-s windows), and the cross correlation is utilized to generate virtual samples for enlarging the number of the training samples. For instance, a sample of the original training dictionary $D^{origin}$ and a virtual sample could be regarded as two samples for a class when both of them are labeled the same class. To better utilize the cross correlation, increasing virtual samples is based on double-length windows. Step 1 and Step 2 of this method are illustrated in Figure 7a and Figure 9 respectively, and the main steps could be summarized as follows.

Step 1: Converting the original training dictionary $D^{origin}$ and its supplemental dictionary $D^{supply}$ to the vectors in the same way as the method of increasing virtual features based on double-length windows.

Step 2: Using the gradient descent algorithm to obtain a virtual sample matrix *S* and vertically concatenate it with $D^{origin}$ to generate a new synthetic training dictionary $D^{synthetic}$.

We should note that this method adds the virtual samples for every activity classes, and the virtual sample matrix *S* has a same structure as $D^{origin}$ including the same arrangement of their features. In addition, the way of obtaining the virtual sample matrix *S* is the same as the way of generating the virtual feature matrix *F* in the method of increasing virtual features based on double-length windows, because they both aim to search a virtual dictionary matrix which inherits the properties of double-length windows. However, the core ideas of them are entirely different. The method of increasing features attaches importance to the different characterizes between the same features but the method of increasing samples confirms the similarities between the same classes from double-length windows. Therefore, as can be seen in the Step 2 of two methods, the original training dictionary $D^{origin}$ is concatenated with the new synthetic dictionary $D^{synthetic}$ horizontally in the former method but vertically in the latter method.

### 4.4. The Method of Increasing Features and Virtual Samples

**Figure 10:**
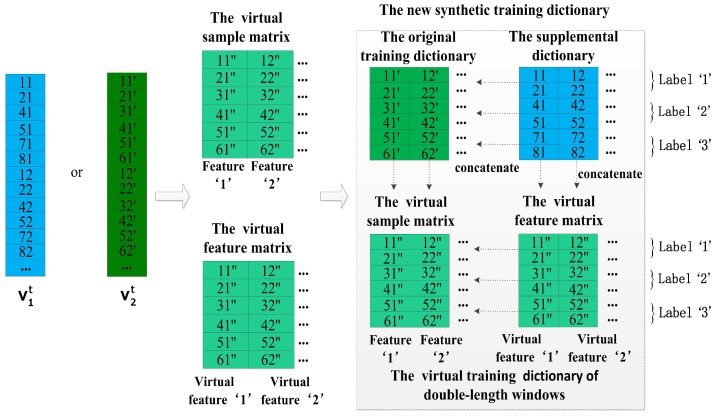
The method of increasing features and virtual samples: Steps 2–4.

Finally, we seek an effective solution for the combination of the method of increasing features and the method of increasing virtual samples. Undoubtedly, all of above methods of increasing features could be utilized in this solution. After a comprehensive consideration from performance and convenience of them (described in Section 5.3), we selected the method of double-length window features among them. Therefore, the key of this solution is in how to utilize the similarity to build a virtual dictionary for a training dictionary of double-length windows, which are generated by the method of double-length window features. Figure 7a and Figure 10 illustrate Step 1 and Steps 2–4 of the method respectively, and the main steps are described as follows:

Step 1: Converting the original training dictionary $D^{origin}$ and its supplemental dictionary $D^{supply}$ to the vectors $v_{1}$ and $v_{2}$ as shown in the Figure 7a.

Step 2: Concatenating $D^{origin}$ with $D^{supply}$ horizontally to obtain a training dictionary of double-length windows.

Step 3: Using gradient descent algorithm to obtain a virtual sample matrix *S* and a virtual feature matrix *F* with above two vectors $v_{1}$ and $v_{2}$, and concatenating the virtual sample matrix *S* with the virtual feature matrix *F* horizontally to generate a virtual training dictionary of double-length windows corresponding to above training dictionary of double-length windows.

Step 4: Concatenating the training dictionary of double-length windows with the virtual training dictionary of double-length windows vertically to generate the final synthetic training dictionary $D^{synthetic}$.

In this method, the virtual sample matrix *S* and the virtual feature matrix *F* are approximately equal to $D^{origin}$ and $D^{supply}$, respectively, so the virtual training dictionary of double-length windows is approximately close to the training dictionary of double-length windows. Lastly, the final $D^{synthetic}$ would be very helpful for complex activity classification.

### 4.5. Analysis and Advantages of the Proposed Methods

In this subsection, we provide the analysis and advantages of above our proposed methods. Because multi-training dictionaries (e.g., $D^{4s}$, $D^{6s}$, $D^{8s}$ and $D^{10s}$) are derived from the same data source which is sampled by users per second as shown in Figure 1, these training dictionaries are correlated with each other in the time domain. Meanwhile, they own individual characteristics due to the different window lengths. The illustration of our experiment 1 described in Section 5.3, also confirms this phenomenon. For complex activity recognition, the effective training samples are very valuable, but sampling too many features would be time- and resource-consuming [12,13]. Therefore, we regard all features of two training dictionaries as different features and utilize their individual characteristics to generate virtual features, so we horizontally concatenate the original training dictionary with another training dictionary or a virtual feature matrix in the above increasing feature methods. In addition, for utilizing the temporal correlation to generate virtual samples, we vertically concatenate the original training dictionary with a virtual sample matrix in the above increasing sample methods. Due to the strong expressive power of the deep learning of DeepMap+, the increased virtual features and samples are beneficial to learn a good representation of the new synthetic training dictionary $D^{synthetic}$ for complex activity recognition.

In summary, our proposed methods have several advantages, as follows. Firstly, all of them are simple and easy to perform. Secondly, the methods do not merely increase the similarities between the same classes but also enhance the difference between different classes from multi-length windows. Furthermore, the methods are beneficial to obtain more detailed information on human hand gestures. Last but not least, our proposed methods are not limited to complex activity recognition, and it can also be applicable to other pattern recognition problems.

## 5. Model Robustness and Comparisons

In this section, we perform the following experiments that investigate the benefit of our proposed methods to DeepMap+ for recognizing high-level indoor semantics with collected large datasets from two users at Wal-Mart.

### 5.1. Data Collection

To validate our proposed methods conveniently, smartphones are used as wearable devices and equipped on the wrists of users for collecting wrist-worn data. Each user carried a Samsung Galaxy S4 smartphone (Samsung Electronics, Suwon, Korea). In our experiments, two users both are forced to use 23 indoor semantics at a Wal-Mart supermarket, and all of indoor semantics and the corresponding activities are illustrated in detail in Table 1. Then, we note that the different durations of indoor semantics utilized by two users result in the difference of sampling number. User 1’s series consist of about 110 min of sensor data collection, and User 2’s series merely consist of about 80 min. Thus, we have received 6691 and 4813 records from sensors on the wrist location of the two users, respectively. The records containing calculated feature streams are broken up into successive sliding windows respectively and there is a 50% overlap. This data has been appended with the corresponding label, and then we made a 75%–25% uniform random split of it to form the train and test datasets for each user respectively. An advanced activity recognition system A-Wristocracy [13] can also be utilized for inferring indoor semantics with our proposed high-level indoor semantic inference. A-Wristocracy is built on single-length windows (such as 2-s or 4-s windows) and trained with an original training dictionary, and DeepMap+ is built on multi-length windows and trained with a new synthetic training dictionary generated by our proposed methods. In this section, A-Wristocracy is denoted by single-length window method. To validate the performance of our proposed methods in DeepMap+, we compare DeepMap+ using our proposed methods with A-Wristocracy and evaluate the effect of varying the lengths (4, 6, 8, 10, 12, 14 s) of windows in various scenarios.

### 5.2. Experimental Setup

The training procedure of DNN in DeepMap+ is implemented in Python and leans upon the Theano deep learning library. We use a multi-layer feed forward artificial neural network, which is learned with stochastic gradient descent relying on back-propagation with two hidden layers. During experiments, we set the training epochs to 1000, learning rate to 0.2, and batch size to 10. The data of all windows is normalized because input normalization greatly impacts the performance of the DL model. In particular, we adopt two techniques to improve DNN fine-tuning. The first technique is the choice of activation functions, we try the ReLU function which has higher convergence speed than the other often used functions (sigmoid, Hyperbolic tangent, etc.). The second is the dropout technique [32] used as a regularization method to address over-fitting during the training process of DL.

Table 2 provides the architecture of DNN in DeepMap+. In these experiments, we do not try to figure out the approximately required number of units in each of the two hidden layer because different training dictionaries give rise to different approximately required numbers. This is not our research objective. The number of units in two hidden layers is set to 100 and 300, respectively. In particular, our experimental sets can protect the fairness of simulation absolutely so that the setup of A-Wristocracy is as the same as DeepMap+. In order to report accurate experiment results, all experiments are using 5-fold cross-validation in this paper.

### 5.3. High-Level Indoor Semantic Classification

**Figure 11:**
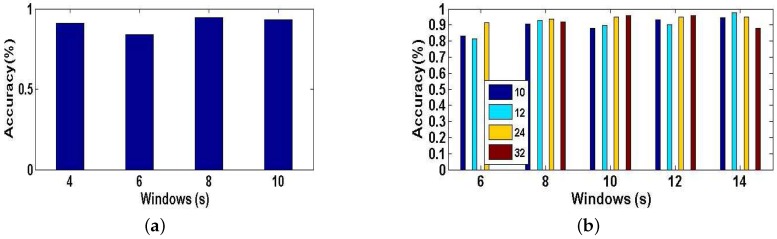
(**a**) Accuracy of the baseline model tested with different training dictionaries from four single-length windows; (**b**) The impact of the length *K* of features set on classification accuracy of our system using the method of increasing virtual features based on multi-length windows.

**Figure 12:**
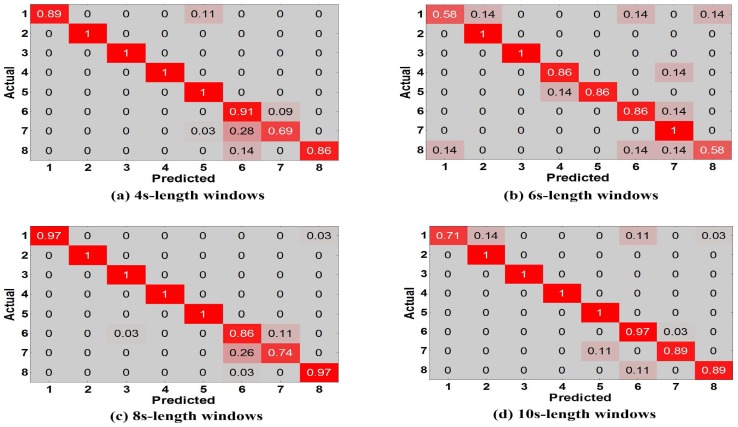
Confusion matrices vs. different lengths of windows.

Experiment 1 is performed for explaining why we have an innovation idea to enhance complex activity classification in this paper. We perform the baseline model [13] trained and tested with different training dictionaries based on single-length windows (4, 6, 8, 10 s) respectively. From User 2’s dataset, we randomly select eight types of indoor semantics to classify. Figure 11a shows the tested accuracy of the baseline model. A notable finding is that the tested accuracy is varied as we train and test the model with different training dictionaries, because different lengths of windows have a significant influence on the complex activity recognition which could affect the final results of high-level indoor semantic inference. Figure 12 illustrates the confusion matrices of test predictions with different lengths of windows. Examining the confusion matrices, we also observe that each different-length window has its specific characteristics. The model may have stronger identification ability for some activities but have lower accuracy for the same activities as the length of windows varies, and similarly the model may have lower identification ability for other activities but have higher accuracy for the same activities as the length of windows changes. For example, the model can achieve a higher accuracy with 100% using the training dictionary based on 6-s windows than the one have a accuracy with 74% based on 8-s windows for the 7th indoor semantic recognition but the exact reverse is the case for the 8th indoor semantic. Meanwhile, there exist strong correlations between the different lengths of windows in the time domain as the model with windows of different lengths achieves all good or poor performance for some specific indoor semantics such as 2nd and 1st indoor semantic, etc. Reasonably utilizing above characteristics and cross correlations is particularly beneficial for DeepMap+ to discover hidden patterns of high-level complex activities. Therefore, it is important to explain that we have the idea of strengthening DeepMap+’s recognizability through: (1) utilizing the multi-length window framework instead of the conventional single-length window framework; and (2) proposing several methods to utilize the characteristics and cross correlations for increasing virtual features and samples.

The Experiment 2 is illustrated the impact of the dimensionality *K* of the synthetic training dictionary generated by the method of increasing virtual features based on multi-length windows for classification accuracy of our system, and DeepMap+ recognizes 23 high-level indoor semantics described in the Table 1. As we describe this method in Section 4.2.3, the length of each features set denotes the number of virtual features generated by PCA. A new synthetic training dictionary is a features set, and we select four different lengths of K (10, 12, 24, 32) of the features set randomly. In Figure 11b, we plot the tested accuracy of DeepMap+ which is trained with new synthetic training dictionaries of K of four different dimensionalities. We can see that the longer length of features set is a double-edged sword. That is because each single-length windows have own characteristic, and the synthetic training dictionary obtained by this method blends all characteristics of multi-length windows and recombines them to extract a new virtual features set, and it may cause data inconsistency even against complex activity recognition and the indoor semantic inference. Therefore, it affirms that the set of virtual features is a significant prior knowledge to this method.

**Figure 13:**
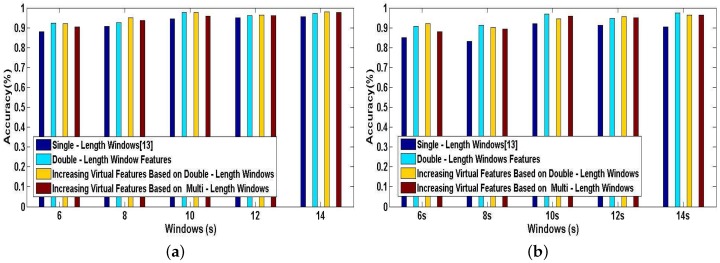
The tested accuracy of the baseline model trained with an original dictionary and our system trained with new synthetic training dictionaries generated by three types of increasing feature methods. (**a**) User 1; (**b**) User 2.

All of the following experiments are performed to validate the performance of our proposed methods in DeepMap+ and recognize 23 high-level indoor semantics. Figure 13a,b illustrates the tested accuracy of A-Wristocracy and DeepMap+. A-Wristocracy is trained with an original training dictionary based on single-length windows and DeepMap+ is trained with new synthetic training dictionaries generated by the method of double-length window features, the method of increasing virtual features based on double-length windows, and the method of increasing virtual features based on multi-length windows. A first observation is that the classification accuracy is more or less improved by above methods through increasing features compared to single-length windows [13] for User 1 and User 2. The reason is that the synthetic training dictionary obtained by above methods of increasing features can better represent the characteristics of complex hand gestures and help DeepMap+ to distinguish the indoor semantics compared to an original training dictionary. The second significant observation is that the performance leaders are the method of double-length window features and the method of increasing virtual features based on double-length windows, respectively. Therefore, considering the performances and applications of above increasing feature methods, the method of double-length window features and the method of increasing virtual features based on double-length windows are the better schemes since neither of them would need any prior knowledge.

**Figure 14:**
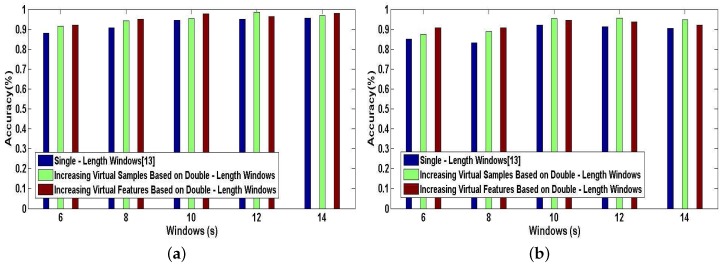
The tested accuracy of the baseline model trained with an original dictionary and our system trained with new synthetic training dictionaries generated by the method of increasing virtual features and the method of increasing virtual samples. (**a**) User 1; (**b**) User 2.

Figure 14a,b shows the tested accuracy of A-Wristocracy [13] and DeepMap+. A-Wristocracy is trained with an original training dictionary based on single-length windows and DeepMap+ is trained with new synthetic training dictionaries generated by using the method of increasing virtual samples and the method of increasing virtual features based on double-length windows respectively. From Figure 14a,b, it is evident that both of our proposed methods outperform the baseline method. Furthermore, for User 1 and User 2, the results of increasing virtual samples are better than increasing virtual features in some cases, but in other cases the results are in contrast. Therefore, we also draw an additional conclusion that increased virtual samples and increased virtual features respectively derived from cross correlations between the same classes and different characteristics between the same features from double-length windows are both beneficial for complex activity recognition, and it would be hard to decide which is the better one for high-level indoor semantic inference.

**Figure 15:**
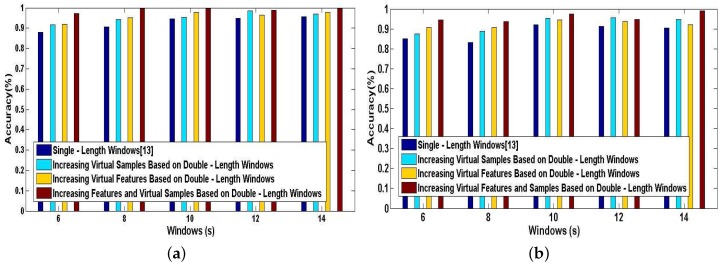
The tested accuracy of the baseline model trained with an original dictionary and our system trained with new synthetic training dictionaries generated by the method of increasing features and virtual samples, the method of increasing virtual features, and the method of increasing virtual samples. (**a**) User 1; (**b**) User 2.

**Figure 16:**
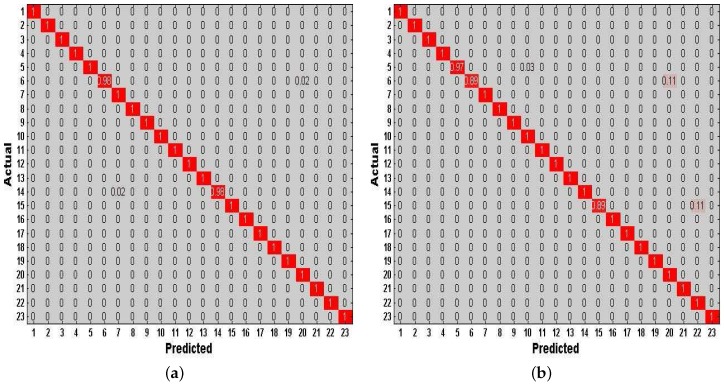
Confusion matrices of test predictions by our system trained with the new synthetic training dictionary generated by the method of increasing features and virtual samples with {10 s, 8 s} windows. (**a**) User 1; (**b**) User 2.

Finally, we investigate the effect of combining the method of increasing features with the method of increasing virtual samples in our DeepMap+. To evaluate the performance of this combined method in DeepMap+, we take it compared with A-Wristocracy [13], DeepMap+ only using the method of increasing virtual samples, and merely using the method of increasing virtual features. Figure 15a,b illustrates the tested accuracy of them for 23 high-level indoor semantic classification. DeepMap+ with the method of increasing features and virtual samples achieves the highest discrimination accuracy at all lengths of windows for User 1 and User 2. Figure 16a,b shows the confusion matrices associated to DeepMap+, which is trained with the new synthetic training dictionary obtained by the method of increasing features and virtual samples based on the double-length {10 s, 8 s} windows. The experiment confirms that the method of increasing features and the method of increasing virtual samples can have a good co-cooperation effect in the field of high-level indoor semantic inference, and DeepMap+ with it achieves the high accuracy 99.6% and 97.52% for User 1 and User 2, respectively.

Above experiments are performed for verifying the classification performance of DeepMap+ with our proposed methods. All of Figure 13, Figure 14, Figure 15 and Figure 16 can confirm the significance of the number of the effective features and samples. Because total samples of all activities and the samples of each activity for User 1 are more abundant than for User 2, we can notice that DeepMap+ and A-Wristocracy [13] have achieved higher recognition accuracies for User 1 than for User 2 in all experiments. Furthermore, DeepMap+ with all of our proposed methods can outperform A-Wristocracy [13] due to the extra virtual features and samples.

### 5.4. System Efficiency

**Figure 17:**
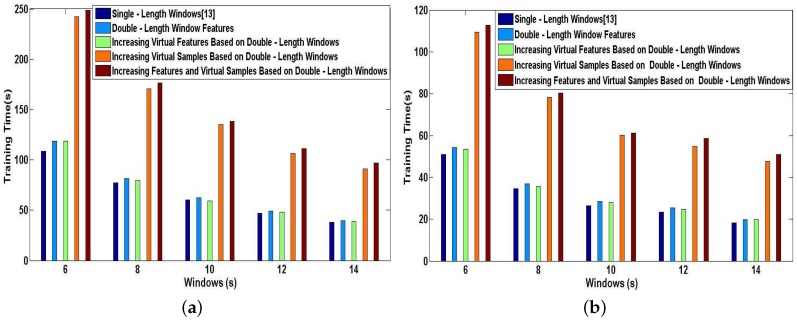
Training time of DNN: the baseline method vs. our proposed methods. (**a**) User 1; (**b**) User 2.

**Figure 18:**
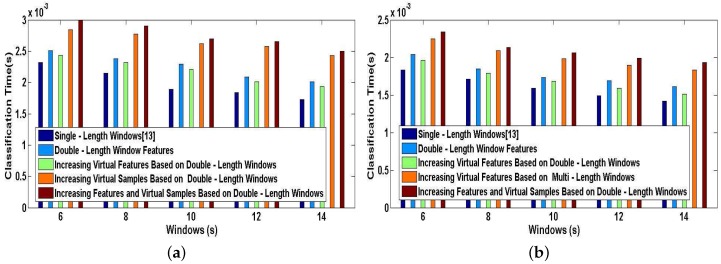
Classification time of DNN: the baseline method vs. our proposed methods. (**a**) User 1; (**b**) User 2.

We now report a series of experiments that demonstrates the efficiency of our proposed methods applied in DeepMap+. We perform the simulations for User 1 and User 2 on a desktop equipped with an Intel Core i7-4770 running at 3.4 GHz and 16-GB RAM. In Figure 17a,b, we plot the training time characteristics for DeepMap+ using our proposed methods and A-Wristocracy [13] using single-length windows. As can be seen, the time cost of the supervised training process may be more or less increased by our proposed methods. The training time of DeepMap+ with the method of increasing virtual samples or the method of increasing features and virtual samples nearly doubles the cost time of A-Wristocracy, and DeepMap+ using the method of double-length window features and the method of increasing virtual features only spends a little more time than A-Wristocracy. Therefore, the above two methods of increasing virtual samples have the lowest execution speed because a greater number of samples puts heavy computation burdens on the learning process of DNN algorithm. This opinion can be also confirmed by comparing the Figure 17a,b, and we notice that DeepMap+ and A-Wristocracy need to learn more samples result in their lower execution speed for User 1. Another important observation is that above increasing feature methods still retain high efficiency compared to above increasing sample methods, although the dimensionality of the training dictionary is added for DNN learning. Figure 18a,b plots the classification time characteristics for DeepMap+ using our proposed methods and A-Wristocracy using single-length windows. We note that the difference of efficiency is minor using above five methods because the tested samples are not big data. Overall, the efficiency of them is enough satisfied to run in real-time when the tested data is not large.

In conclusion, complex indoor activity classification and high-level indoor semantic inference are advanced by DeepMap+ using our proposed methods, although the execution time of our system may be improved more or less. However, we think that the loss of time is negligible for building DeepMap+ which has the offline self-learning capability. Furthermore, DeepMap+ has the optimal efficiency when utilizing the method of increasing virtual features based on double-length windows, and it has the best classification performance when equipping with the method of increasing features and virtual samples based on double-length windows.

## 6. Conclusions

In this work we proposed the DeepMap+, an automatical inference system based on DL with wrist-worn device sensing for recognizing abundant high-level indoor semantics using complex human activities. In addition, we presented a multi-length window framework instead of the conventional single-length window framework to greatly enrich the training dictionary source of DeepMap+. In our opinion, the multi-length window framework has an overwhelming advantage because there exist cross correlations between the same classes and the characteristics between the same features from windows of different lengths. We found that the correlations and the characteristics are beneficial to increase virtual samples and features for finely capturing hand gestures. Based on this, we designed several methods about increasing virtual samples and features to generate a synthetic training dictionary which can contain more robust representations learned by DeepMap+. We believed that our proposed methods based on the multi-length window framework can be also applied to recognition of other patterns. Finally, DeepMap+ has been shown to classify 23 high-level indoor semantics for two users at a Wal-Mart supermarket. The experimental results validated that DeepMap+ with our proposed methods had higher average tested accuracy compared to A-Wristocracy [13]. The next phase of our studies is to exploit the popular WiFi signals for recognizing complex human activities and high-level indoor semantics. The best advantage is that it is device-free for all users if we apply channel state information (CSI) measurements to the fields of human activity recognition and indoor semantic inference.

## Figures and Tables

**Table 1:** The detailed description of complex activities.

ID	Layer-1: Complex Activity	Layer-2: Public Facility	Layer-3: Functional Zone	The Detailed Description of the Activity
1	Opening a one-door soda fountain	A one-door soda fountain	The food area	Subject opens the door of the soda fountain with
				one hand, then removes the goods and closes the door.
2	Grabbing a cling wrap	A cling wrap supply shelf	The food area	Subject grabs a cling wrap, then pulls it out
				and tears it off from the shelf.
3	Bagging bulk food	A bulk food display shelf	The food area	Subject picks up the bulk food with a hand and puts them
				into a freshness packet which is grasped by another hand.
4	Opening a two-door upright freezer	A two-door upright freezer	The bread section	Subject opens the doors of the upright freezer with two
				hands, then picks up the goods and closes the door.
5	Taking a bread or sandwich with a food clip	A cake counter	The bread section	Subject pulls out the drawer of bread counter, then picks
				up the bread with a clip and finally pushes back the drawer.
6	Opening a horizontal freezer	A horizontal freezer	The meat section	Subject pushes the freezer door open with the palm of the hand downward,
				then picks up the meat and pulls back the door until it is closed.
7	Selecting a bottle of wine	A wine cabinet	The wines section	Subject grasps the bottleneck with one hand and holds the bottom
				of the bottle with the other hand, then rotates the bottle body.
8	Filling rice into storage bag with a measured cup	A rice display shelf	The mixed grain rice section	Subject picks a measured cup and
				ladles a cup of rice into a storage bag.
9	Picking over an apple	A fruit and vegetable storage shelf	The section of fruits and vegetables	Subject picks up the fruit and the wrist rotates
				so that his palm turns from downward to upward.
10	Trying on a trousers	A fitting room	The trousers section	Subject takes off his trousers, and then puts another pair of trousers on.
11	Trying on a shoe	A shoe display shelf	The shoes section	Subject bends down to untie the shoelace, then takes off the shoe,
				next puts on another shoe and ties the shoelace.
12	Trying on a jacket	A jacket display shelf	The clothes section	Subject takes off his jacket, and then puts another jacket on.
13	Getting a cup of water from a drinking fountain	A drinking fountain	The drinking fountain	Subject takes a cup at the front of the machine, then presses
				down the button and waits 2–3 s, finally takes away the cup.
14	Touching a cotton goods like mattress	A bedding articles display shelf	The area of living goods	Subject lightly touches and beats the cotton
				goods with a hand to feel the softness of it.
15	Browsing a book or notebook	A book display shelf	The area of cultural and sports goods	Subject holds a book or a notebook with both hands and flips through its pages.
16	Writing	A pen display shelf	The area of cultural and sports goods	Subject picks up a pen and writes several characters.
17	Examining a drum washing machine	A drum washing machine	The Electrical area	Subject bends over and opens the door of drum washing machine from the upper
				right, then examines the internal structure and closes the door.
18	Putting goods on the checkout counter	A checkout counter	The checkout counter	Subject picks up the goods from the shopping basket
				and puts them on the checkout counter.
19	Opening a door of emergency exit	A emergency exit	The emergency exit	Subject pushes forward the pole of the emergency exit and opens the door.
20	Heating food with a microwave oven	A utilizable microwave oven	The service counter	Subject presses down the door open button, then takes into the foods and closes
				the door, next spins the button to turn on the heat.
21	Washing hands	A tap	The rest room	Subject turns on the tap, and scrubs his hands repeatedly.
22	Standing in an escalator	An escalator	The escalator	Subject holds the handrail of escalator and stands motionless.
23	Walking in the stairs	A stairs	The stairs	Subject walks in the stairs.

**Table 2:** DNN architecture.

Total Layers	Hidden Layers	Units in the First Hidden Layer	Units in the Second Hidden Layer
4	2	100	300
